# Comparative transcriptome analysis reveals distinct gene expression profiles in *Brachypodium distachyon* infected by two fungal pathogens

**DOI:** 10.1186/s12870-021-03019-0

**Published:** 2021-06-30

**Authors:** Gengrui Zhu, Chengyu Gao, Chenyu Wu, Mu Li, Jin-Rong Xu, Huiquan Liu, Qinhu Wang

**Affiliations:** 1 State Key Laboratory of Crop Stress Biology for Arid Areas and College of Plant Protection, Northwest A&F University, Yangling, 712100 Shaanxi China; 2 Department of Botany and Plant Pathology, Purdue University, West Lafayette, IN 47907 USA

**Keywords:** *Fusarium graminearum*, *Magnaporthe oryzae*, *Brachypodium distachyon*, Plant-fungal interaction, RNA-seq

## Abstract

**Background:**

The production of cereal crops is frequently affected by diseases caused by *Fusarium graminearum* and *Magnaporthe oryzae*, two devastating fungal pathogens. To improve crop resistance, many studies have focused on understanding the mechanisms of host defense against these two fungi individually. However, our knowledge of the common and different host defenses against these pathogens is very limited.

**Results:**

In this study, we employed *Brachypodium distachyon* as a model for cereal crops and performed comparative transcriptomics to study the dynamics of host gene expression at different infection stages. We found that infection with either *F. graminearum* or *M. oryzae* triggered massive transcriptomic reprogramming in the diseased tissues. Numerous defense-related genes were induced with dynamic changes during the time course of infection, including genes that function in pattern detection, MAPK cascade, phytohormone signaling, transcription, protein degradation, and secondary metabolism. In particular, the expression of jasmonic acid signaling genes and proteasome component genes were likely specifically inhibited or manipulated upon infection by *F. graminearum*.

**Conclusions:**

Our analysis showed that, although the affected host pathways are similar, their expression programs and regulations are distinct during infection by *F. graminearum* and *M. oryzae*. The results provide valuable insight into the interactions between *B. distachyon* and two important cereal pathogens.

**Supplementary Information:**

The online version contains supplementary material available at 10.1186/s12870-021-03019-0.

## Background

Wheat and rice are two staple cereal crops that feed humans. However, their production is frequently affected by many fungal pathogens [1]. Among them, *Fusarium graminearum* and *Magnaporthe oryzae* are two of the most devastating plant pathogenic fungi, mainly causing *Fusarium* head blight (FHB) in wheat and blast disease in rice, respectively [2, 3]. FHB epidemics occur in many countries and cause a severe reduction in grain quantity and quality [4, 5]. *F. graminearum* infects the kernel of wheat, thus leading to a direct yield loss. In addition, the mycotoxins deoxynivalenol (DON) and zearalenone (ZEA) that reside in the wheat grains are dangerous to humans and animals, resulting in further damage to the quality of wheat products [4, 6]. Rice blast is also a global disease that threatens rice production [7]. In China, epidemics often cause yield losses of 40–50% or even up to 100% in severely infected areas [8]. Yield losses range from 50 to 85% in the Philippines [8]. It is estimated that the rice destroyed by this disease is sufficient to feed 60 million people, leading to an economic loss of $66 billion annually [9]. In addition, some lineages of *M. oryzae* are responsible for the recent wheat blast outbreaks in South America and Southeast Asia [10–12].

The discovery, utilization, and deployment of resistant cultivars is an effective approach to control these diseases [13, 14]. Resistant germplasm for FHB is rare in wheat, and only 155 quantitative trait loci (QTL) that contribute to FHB resistance have been identified [5, 15, 16]. Sources of resistant germplasm for rice blast are available, but they are frequently overcome due to the rapid evolution of new pathotypes of the pathogen [8, 17]. Therefore, it is essential to determine the mechanisms of pathogen infection and host defense. To understand host defense responses, many transcriptomic analyses have been conducted in plants infected with *F. graminearum* or *M. oryzae* [18–33]. For example, although a majority of wheat cultivars are susceptible to *F. graminearum* infection, the gene expression profiles in the cold-killed and living wheat heads are obviously distinct and suggest that the host defense responses actively suppress fungal growth during infection [18]. In line with this evidence, many transcriptomic studies showed that genes related to oxidative burst, MAPK signaling, hormone biosynthesis, transcription, secondary metabolism, and other defense associated proteins were altered upon infection [19–25]. These defense responses are also observed in *M. oryzae* infection [26–32] and many other plant-pathogen interactions [33].

To use the host defense mechanisms for resistance breeding, it is important to know which type of defenses were most responsive in the hosts and which were generally fired by the pathogens. However, our knowledge of these differences in host defenses is very limited. Considering wheat and rice have distinct habits, genome size, and gene number, a direct comparison of their differences in host defenses is difficult and may lead biased conclusion. Therefore, developing an efficient model for dissecting these differences is required. A model of cereal species, *Brachypodium distachyon*, is susceptible to many important cereal pathogens, including *F. graminearum* and *M. oryzae*, and serves as a model for studying cereal diseases [34]. Disease development of *B. distachyon* caused by *F. graminearum* and *M. oryzae* is highly similar to that on wheat [35] and rice [36, 37], respectively. Furthermore, transcriptional analyses showed that the expression of the defense-related genes is also similar to those in wheat [38] and rice [34, 36], respectively. In this study, we employed two different pathosystems, *B. distachyon*-*F. graminearum* and *B. distachyon*-*M. oryzae*, and performed comparative transcriptomics to study the dynamics of host gene expression upon infection. Our analysis showed that, although many similar defense responses have been observed in *B. distachyon* challenged by *F. graminearum* or *M. oryzae*, the expression programs and some specific defense responses are very different in these two pathosystems.

## Results and discussion

### Distinct global gene expression of *Brachypodium* infected by two cereal fungi

**Fig. 1:**
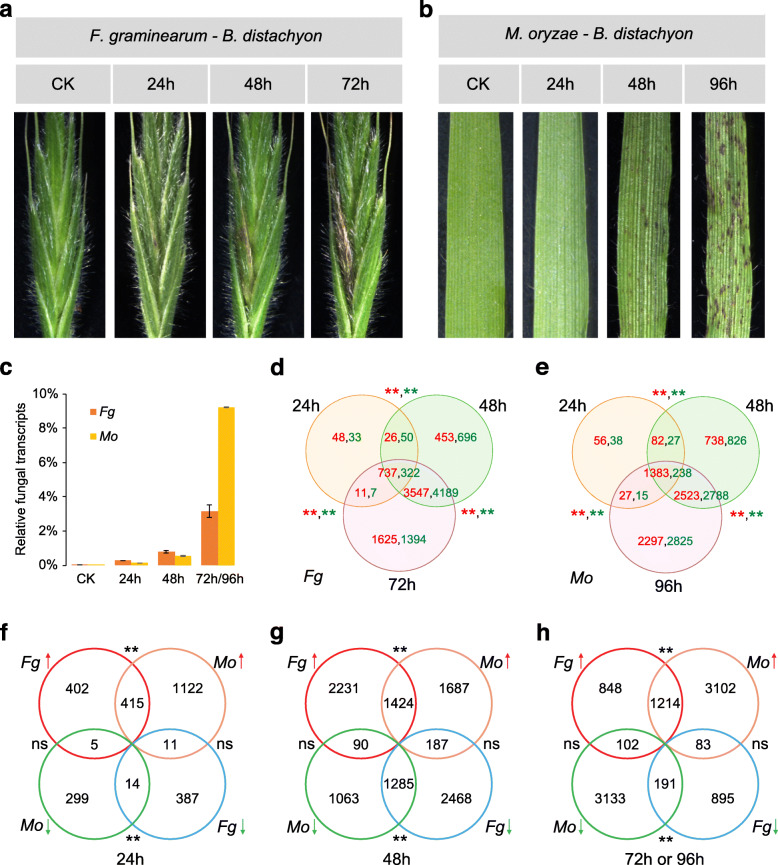
Disease development and differentially expressed genes of *B. distachyon* infected by *F. graminearum* or *M. oryzae*. (**a**) Disease development of *B. distachyon* spikes infected by *F. graminearum*. (**b**) Disease development of *B. distachyon* leaves infected by *M. oryzae*. (**c**) The relative fungal transcript levels during *B. distachyon* - fungal interaction. (**d**) Venn diagram showing the common and differential transcriptional responses of up-regulated (in red) and down-regulated (in green) genes of *B. distachyon* spikes infected by *F. graminearum* at different time points. (**e**) Venn diagram showing common and differential transcriptional responses of up-regulated (in red) and down-regulated (in green) genes of *B. distachyon* leaves infected by *M. oryzae* infection at different time points. (**f-h**) Venn diagram showing the number of up-regulated (red arrows) and down-regulated (green arrows) genes of *B. distachyon* inoculated with *F. graminearum* and *M. oryzae*, respectively, at 24 h (**f**), 48 h (**g**), and 72/96 h (**h**). The statistical significances of the overlapped genes were analyzed by hypergeometric tests. “**”, *P* < 0.01; “ns”, not significant

To investigate the transcriptional dynamics during *Brachypodium*-fungal interactions, we performed RNA-seq analysis on *B. distachyon* infected by two cereal pathogens: *F. graminearum* and *M. oryzae*. In nature, *F. graminearum* and *M. oryzae* mainly infect the spike and leaf of wheat and rice, respectively. We therefore employed the spike and leaf of *B. distachyon* to mimic their infections. Based on the lesion development (Fig. 1a-b) and infection progress [35–37], three stages (24 h, 48 h, and 72 h) of *B. distachyon* spikes infected by *F. graminearum*, and three stages (24 h, 48 h, and 96 h) of *B. distachyon* leaves infected by *M. oryzae*, were collected. Together with the mock of *B. distachyon* spike and leaf, eight samples with three biological replicates were sequenced in these two pathosystems (Table S1).

When mapping to fungal genomes (Fig. 1c, Table S1), only a small number (less than 1%) of fungal mRNA reads were found in the early stages of infection (24–48 h). In the later stage of infection (72 and 96 h for *F. graminearum* and *M. oryzae*, respectively), substantial fungal mRNA reads were detected (3–10%). The gradual increase in fungal mRNA reads is highly consistent with the lesion development in *B. distachyon*. The dramatic increment of fungal mRNA reads in the later stages suggests that the fungi have absolutely broken plant defenses and switched to necrotrophic growth.

For each RNA-seq library, approximately 81.2% of the reads were mapped to the *B. distachyon* genome (Table S1). Among the 25,532 *B. distachyon* genes, 20,917 (78.8%) and 18,978 (71.5%) expressed genes were detected in spikes and leaves infected by *F. graminearum* and *M. oryzae*, respectively. Multi-dimensional scaling plots (Fig. S1a-b), which illustrate the intrinsic biological variation among samples, showed that samples from the same infection stage were grouped together. Hierarchical clustering (Fig. S1c-d), which was constructed from the distances between the RNA-seq libraries in the experiment, revealed that the gene expression profiles of *B. distachyon* are distinct at different stages following infection by *F. graminearum* and *M. oryzae*.

In comparison to controls, 44.1 and 58.6% of the expressed genes are affected in at least one stage upon infection by *F. graminearum* and *M. oryzae*, respectively. Therefore, approximately half of the *B. distachyon* genes are likely involved in the battle with fungal pathogens, although many genes may be passively up-regulated or down-regulated. Generally, the specific differentially expressed genes (DEGs) for each stage continue to increase with lesion development in both pathosystems (Fig. 1d-e). This is consistent with the colonization of fungi in the host. The majority of DEGs (Fig. 1d-e), especially the DEGs highly specific (≥10 fold) to individual fungi (Table S2), are found in the later stages of infection, suggesting that host physiology is greatly affected and the plant is forced to focus on struggling with the pathogens at those stages.

Examination of the specific DEGs for each pathogen revealed that the mode of gene up-regulation and down-regulation is different in *F. graminearum* and *M. oryzae* infections (Fig. 1f-h). While *F. graminearum*-infected samples have the maximum number of DEGs at 48 h post-inoculation (hpi), *M. oryzae*-infected samples have the maximum number of DEGs at 96 hpi. These may associate with different biotrophic-necrotrophic progress in these two distinct pathogens. Taken together, these data suggest that many genes are affected upon infection, and the global gene expression of *B. distachyon* infected by *F. graminearum* and *M. oryzae* are very different from each other.

### Distinct gene expression program of *Brachypodium* infected by two cereal fungi

**Fig. 2:**
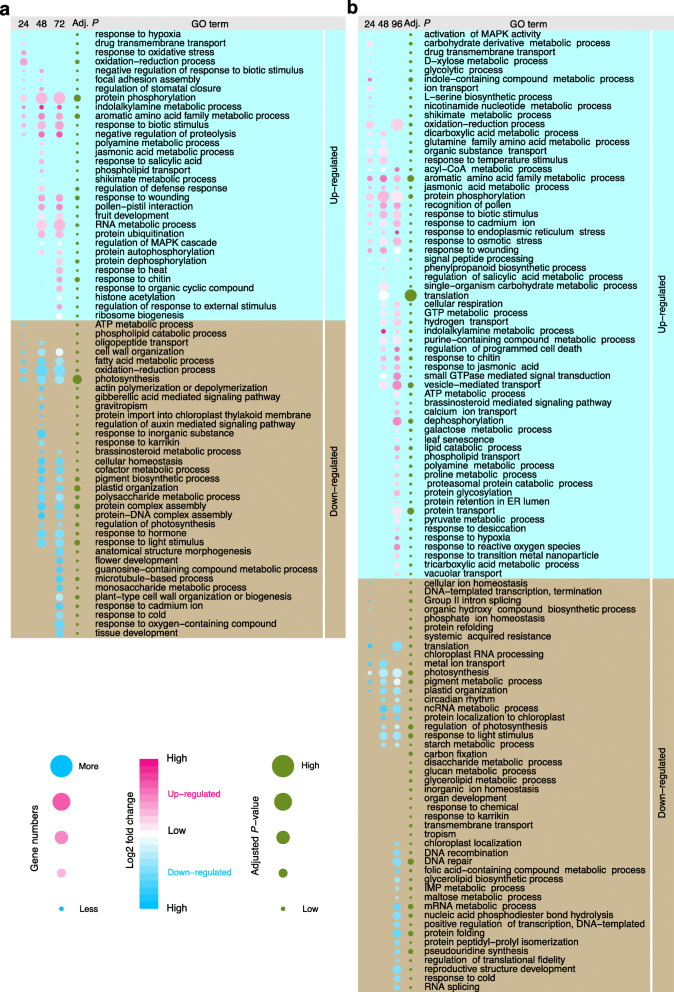
Gene Ontology enrichment analysis of the differentially expressed genes of *B. distachyon* infected by *F. graminearum* (**a**) and *M. oryzae* (**b**). The size of the blue/red circle (in the first three columns) represents the number of the enriched genes, while the color represents the corresponding average log2 fold change. Red and blue circles indicate the up-regulation and down-regulation of the enriched gene, respectively. The adjusted *P*-values are illustrated with the size of the green circles. In (a-b), only adjusted *P*-values less than 0.05 are shown

To compare the functional difference carried by gene expression dynamics, we performed gene ontology (GO) enrichment analysis on the DEGs from *F. graminearum*- and *M. oryzae*-infected samples at different infection time points. Many of the enriched GO terms are associated with plant-pathogen interaction, suggesting that the host gene expression had been greatly affected by pathogen infections, rather than the developmental changes of leaf and spikelet during the sampling. Throughout all the interactions, genes involved in “response to biotic stimulus” are up-regulated and enriched in *F. graminearum*- and *M. oryzae*-infected samples, while genes related to or involved in “photosynthesis” are down-regulated and enriched in *F. graminearum*- and *M. oryzae*-infected samples (Fig. 2). This suggests that *B. distachyon* had switched on plant defense and compromised to basal metabolism during the entire interaction. Similar tradeoffs between photosynthesis and plant defense have also been observed in many other studies [39].

While some of the *B. distachyon* genes were enriched in both fungal infections at the equivalent stages, some were enriched in only one of the interactions (Fig. 2). For example, genes involved in “drug transmembrane transport” were enriched in the early stage of both fungal infections, whereas genes involved in “histone acetylation” and “glutamine family amino acid metabolic process” are enriched solely in *F. graminearum*- or *M. oryzae*-infection, respectively.

Some defense-related genes were up-regulated and enriched in both *F. graminearum*- or *M. oryzae*-infection, but some appeared at a different stage in the other pathosystem (Fig. 2). For example, genes related to “polyamine metabolic process”, “jasmonic acid metabolic process”, and “shikimate metabolic process”, which are known to be involved in plant defenses [40–42], are enriched in both *F. graminearum*- or *M. oryzae*-infection. During the *B. distachyon*-*F. graminearum* interaction, genes involved in the “polyamine metabolic process” are up-regulated at 48 hpi, but they are up-regulated at 96 hpi in *B. distachyon*-*M. oryzae* interaction. While genes involved in the “jasmonic acid metabolic process” are enriched at 48 hpi by *F. graminearum*, they are enriched throughout the *M. oryzae* interaction. Unlike the early stage (24 hpi), which involves genes related to the “shikimate metabolic process” in *B. distachyon*-*M. oryzae* interaction, these genes began to be enriched at 48 hpi in *B. distachyon*-*F. graminearum* interaction.

Therefore, the expression programs of the defense-related genes are distinct during *F. graminearum* and *M. oryzae* infections. It is unclear how the plant orchestrates these different defense programs to deal with various pathogens. However, most likely the different pathogen-associated molecular patterns (PAMPs) are detected by distinct receptors on the cell membrane, thus triggering multiple host defenses. Further characterization of the code of PAMPs detection and the pathways leading to subsequent cascades may be crucial for understanding these defense programs.

### Distinct gene expression network of *Brachypodium* infected by two cereal fungi

**Fig. 3:**
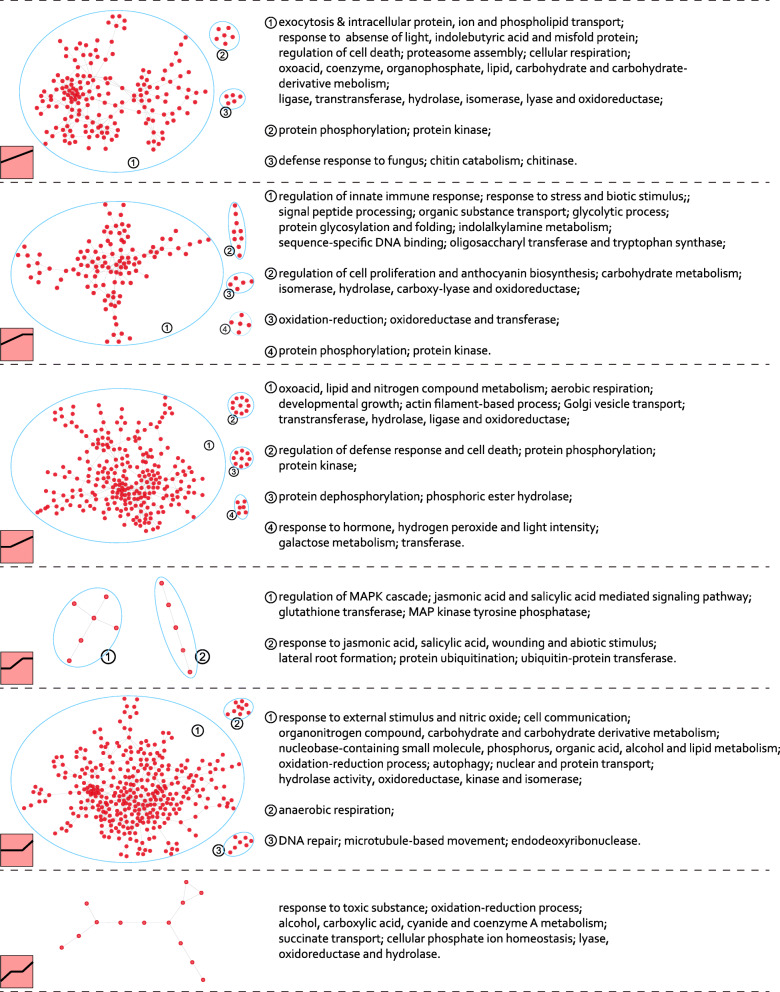
Protein-protein interaction networks of the up-regulated genes in *B. distachyon* infected by *M. oryzae*. The up-regulated genes in each profile were subjected to protein-protein interaction networks analysis. Gene Ontology annotation was used to reveal the function of each submodule of the networks (blue oval). Nodes and edges represent proteins and functional links, respectively

To further understand the dynamic expression pattern of DEGs at different time points, we clustered the DEGs into different model profiles using STEM software [43]. Ten and twelve significant expression profiles were identified from the *F. graminearum*- and *M. oryzae*-infected plants, respectively (Fig. S2). Genes in the same expression profile are potentially co-regulated and more likely to functionally associate with each other [44, 45]. We therefore integrated the clustered gene expression profiles with protein-protein interaction networks. With the help of the STRING database [46], eight from the *F. graminearum*-infected plants and twelve from the *M. oryzae*-infected plants were extracted, and most of them were comprised by several modules (Fig. 3, Fig. S3–5).

Functional annotation of these networks revealed that many of them are associated with plant-pathogen interactions (Fig. 3, Fig. S3–5). For example, during the *B. distachyon*-*F. graminearum* interaction, a series of co-expression modules, such as MAPK signaling, plant hormone signaling, gene transcription, and many plant defense-related responses, were up-regulated (Fig. S3). Interestingly, two modules in plant hormone signaling, exhibited distinct gene expression profiles. The genes related to “regulation of jasmonic acid mediated signaling pathway” continue to upregulation all the time, whereas the genes related to “response to ethylene” stop to upregulation in the later stage (Fig. S3).

During the *B. distachyon*-*M. oryzae* interaction, except the co-upregulation of MAPK signaling and plant hormone signaling related genes, many distinct networks associated with different plant defense responses were also involved (Fig. 3), such as “exocytosis”, “Golgi vesicle transport”, “chitin catabolism”, “regulation of cell proliferation and anthocyanin biosynthesis”, “response to external stimulus and nitric oxide”, “response to toxic substance”, as well as the “regulation of innate immune response”, “regulation of defense response and cell death”, etc. These data suggest that, although many defense-associated networks are overlapped, substantial networks in response to different fungi are distinct. Consistent with the defense programs, this analysis also showed that different biological processes worked in concert to knit the total plant defense.

### MapMan analysis revealed involvement of different pathways in *B. distachyon*-fungal interactions

**Fig. 4:**
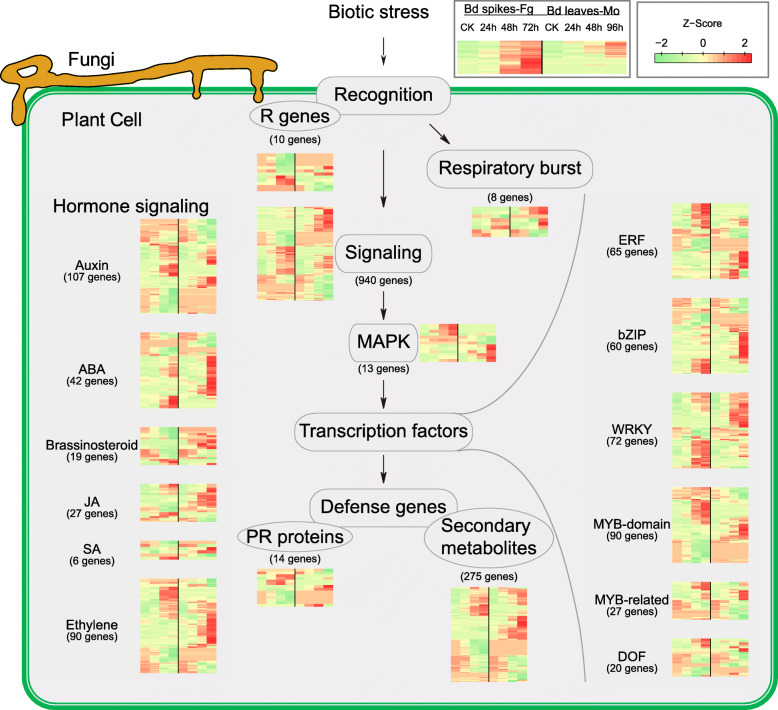
*B. distachyon* defense mechanisms activated during the interaction. In each step, the dynamics of the gene expression are illustrated with a heatmap. For each heatmap, the first four columns are *B. distachyon* spikes inoculated with *F. graminearum* at 0 h, 24 h, 48 h, and 72 h; the second four columns are *B. distachyon* leaves inoculated with *M. oryzae* at 0 h, 24 h, 48 h, and 96 h. The expression levels are mapped to color from green to red, to represent a lower to a higher abundance of the transcripts

To understand the details of *B. distachyon* defense mechanisms that are activated during the interaction, we mapped individual gene expressions into metabolic pathways by using MapMan [47]. Pathway enrichment analysis based on Wilcoxon tests (Table S3) revealed that many genes encoding receptor-like kinases, WRKY transcription factors, or genes involved in ethylene, jasmonate, aromatic amino acid metabolism, protein synthesis or degradation were remarkably up- or down-regulated during both fungal infections. In the *B. distachyon*-*M. oryzae* pathosystem, many additional genes essential for hormone metabolism and secondary metabolism were affected. We therefore examined several major classes of genes responsive to biotic stress (Fig. 4). The results showed that a large number of them were altered during the interactions.

#### Pattern detection

When pathogens initiate contact with the plant, some conserved PAMPs are perceived via many receptor-like kinases (RLKs) and receptor-like proteins in the plasma membrane [48]. According to the MapMan analysis, 454 genes belong to the RLK family. Interestingly, many of the 192 cysteine-rich RLKs (with the DUF 26 domain) genes and LRK10-like RLKs were significantly up-regulated in both *F. graminearum* and *M. oryzae* infections (Table S3–4). Although the roles of cysteine-rich RLKs are unknown in *B. distachyon*-fungal interactions, they were shown to have a positive role in regulating anti-bacterial resistance [49]. The expression levels of some receptor-like kinases (RLK) genes were increased (Table S4), which may allow the generation of a strong danger signal. At the same time, some were suppressed and blocked by *F. graminearum* or *M. oryzae* when infecting *B. distachyon* (Table S4). This result suggests that, during the first encounter, neither the plant nor the pathogen has an advantage. They are both compromised. The behavior of *RLK* genes may be shaped by their co-evolution, since unsuccessful pathogens and plants with inferior fitness may be eliminated by strong constraints from each other.

#### MAPK signaling

Mitogen-activated protein kinase (MAPK) cascades play crucial roles in plant defense against pathogens. Activation of MAPK cascades is one of the earliest responses after the plant senses pathogen-associated molecular patterns (PAMPs) [50, 51]. In turn, a successful pathogen may suppress host MAPK cascades via their armed effectors [52, 53]. During the interaction of *B. distachyon* with these two fungi, different *MAPK* genes were up-regulated at later stages (Fig. 4). This result suggests that different MAPK may be employed by *B. distachyon* to translate pathogen attack signals from the two cereal pathogens and also suggests that these two pathogens have succeeded in suppressing the earliest defense cascades by MAPK, which may further facilitate the infection.

#### Phytohormone signaling

Phytohormones, such as salicylic acid (SA), jasmonate (JA), and ethylene (ET), are major plant hormones that orchestrate plant immunity [54–56]. Consistent with the brief biotrophic phase of *F. graminearum* and *M. oryzae* in infection [57, 58], expression of genes involved in SA signaling seems to have changed very little upon infection. Notably, many genes related to JA and ET signaling were significantly up-regulated (Fig. 4), as observed in the wheat- *F. graminearum* interaction [59]. These data suggest that the JA and ET pathways are critical for responding pathogens like *F. graminearum* and *M. oryzae*.

**Fig. 5:**
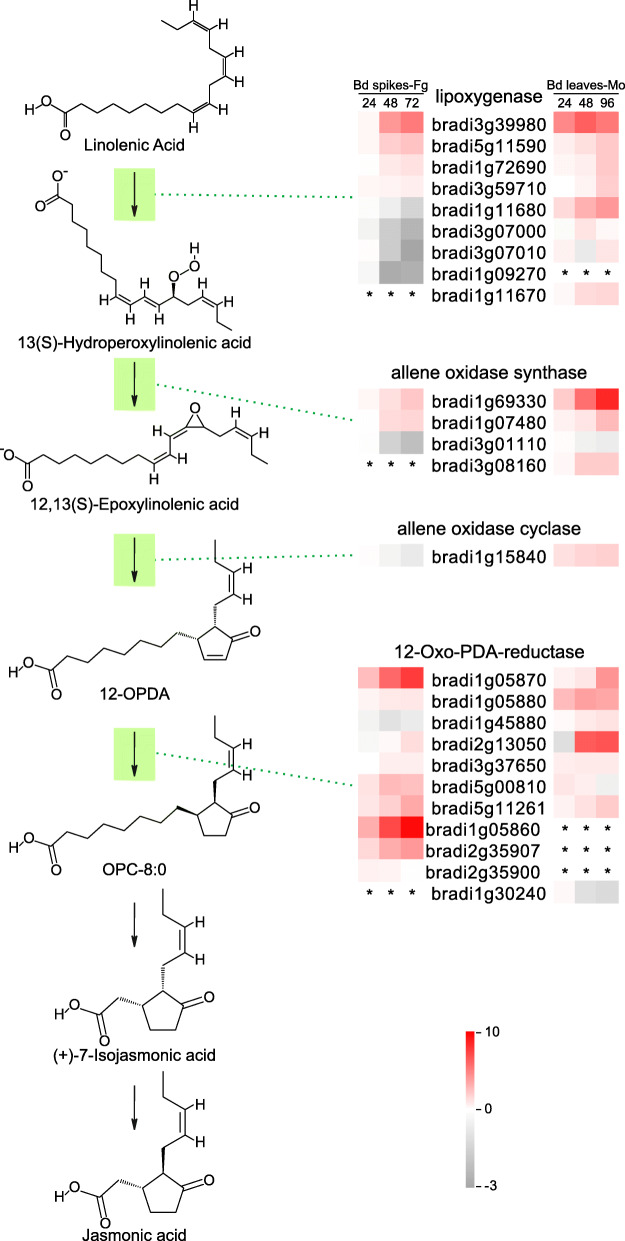
The response of JA biosynthesis genes in *B. distachyon* upon *F. graminearum* and *M. oryzae* infection. The responses of the genes involved in the JA biosynthesis is illustrated with a heatmap. For each gene, the log2 fold change (logFC) level is mapped to color from gray to red, to represent the down-regulation and up-regulation of gene expression. The asterisks indicate no changes in expression

While the number of specifically induced ET-related genes are comparable in *F. graminearum* and *M. oryzae* infection, the number of specifically induced JA-related genes upon *F. graminearum* infection are obviously lower (Fig. 4). Close examination showed that behaviors of JA biosynthesis genes in *F. graminearum*-and *M. oryzae*-infected *B. distachyon* are quite different (Fig. 5). In the first two steps, the number of induced JA biosynthesis genes (*LIPOXYGENASE*, and *ALLENE OXIDASE SYNTHASE*) were consistently lower in *F. graminearum* infection than those in *M. oryzae* infection. In particular, *Bradi1g15840*, which encodes the only allene oxidase cyclase (AOC), was down-regulated during the *B. distachyon*-*F. graminearum* interaction. Such fine-tuning may lead to less 12-OPDA production and an insufficient final level of JA metabolites. Therefore, JA biosynthesis is likely to be suppressed in the *B. distachyon*-*F. graminearum* interaction.

Auxin (AUX), abscisic acid (ABA), and brassinosteroid (BR) have also been shown to be involved in phytohormone signaling [60, 61]. Remarkable differences in AUX- or ABA-responsive gene expression occurred between *B. distachyon* infected with *F. graminearum* and *M. oryzae*, respectively (Fig. 4). Each of the two fungi caused a similar number of specifically up-regulated genes, suggesting different downstream signaling pathways may be activated upon either *F. graminearum or M. oryzae* infection, respectively. This pattern is not observed in BR signaling. Interestingly, we found that some BR-responsive genes that were up-regulated in *M. oryzae*-infection were down-regulated in *F. graminearum*-infection, suggesting that BR signaling is likely to be inhibited during the *B. distachyon*-*F. graminearum* interaction.

#### Transcription factors

Early plant defense signals often result in the activation of downstream transcription factor genes to enhance defense-related gene expression [62]. Many transcription factor genes, such as *ERF*, *bZIP*, *WRKY*, *MYB* genes were up-regulated upon the infection by these two cereal fungal pathogens, respectively [24, 29]. Interestingly, most of the WRKY transcription factor genes that were up-regulated in *B. distachyon*-*F. graminearum* interaction are distinct from the ones up-regulated in *B. distachyon*-*M. oryzae* interaction (Fig. 4). This may be a result of different MAPK signaling pathways employed by the host plant. The distinct MAPK signaling, and distinct up-regulation of WRKY transcription factor genes suggest that *B. distachyon* has evolved different mechanisms to deal with these two different fungi.

#### Secondary metabolism

**Fig. 6:**
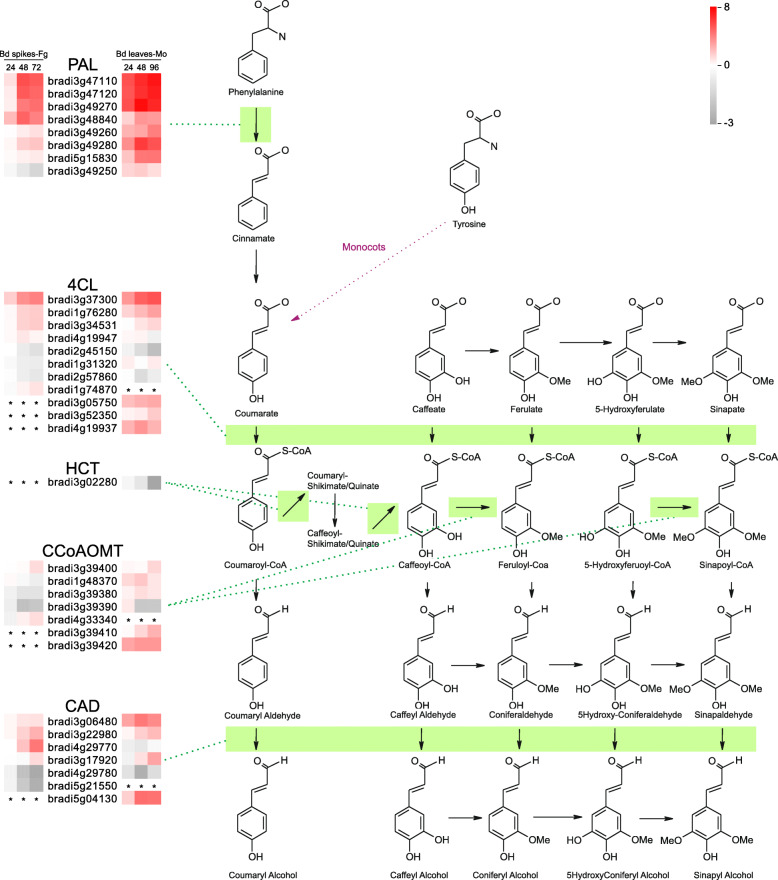
The response of phenylpropanoid metabolism genes in *B. distachyon* upon *F. graminearum* and *M. oryzae* infection. The responses of the genes involved in phenylpropanoid metabolism are illustrated with heatmaps. For each gene, the log2 fold change (logFC) level is mapped to color from gray to red, to represent the down-regulation and up-regulation of gene expression. The asterisks indicate no changes in expression

The final defenses are mediated by many defense-related genes, such as *PR* genes and secondary metabolism genes [63–65]. Genes involved in the metabolism of phenylpropanoid (82), isoprenoid (71), and flavonoid (56) comprise three-quarters of the 275 genes related to secondary metabolism (Table S5). MapMan enrichment showed that phenylpropanoid metabolism genes are induced, while genes involved in isoprenoid and simple phenol metabolism are repressed in the *B. distachyon*-*M. oryzae* interaction (Table S3). Phenylpropanoid metabolism leads to the biosynthesis of lignin and plays an important role in plant defense [66, 67]. In the infected *Brachypodium*, transcriptional changes in this pathway mainly occurred in the genes encoding phenylalanine ammonia lyase (PAL), 4-coumarate CoA ligase (4CL), hydroxycinnamoyl-CoA shikimate/quinatehydroxy cinnamoyl transferase (HCT), caffeoyl-CoA O-methyltransferase (CCoAOMT), and cinnamyl alcohol dehydrogenase (CAD) (Fig. 6), which are known to play different roles in regulation of the resistance to diverse pathogens [67–75]. Many components of the phenylpropanoid pathway are targeted by different effectors [72, 76, 77], suggesting that the pathogens recognize the importance of this pathway and have evolved corresponding strategies to deal with the defense conferred by this pathway. Upon *F. graminearum* and *M. oryzae* infection, many upstream genes of phenylpropanoid metabolism, such as *PALs*, were generally up-regulated, while some of the downstream genes, such as *CADs*, showed diverse expression patterns (Fig. 6). These data suggest that, although plant defense from the phenylpropanoid pathway was initiated, the products that execute the defense were likely to be attenuated by these two cereal fungi.

#### Protein degradation by the ubiquitin-proteasome system

**Fig. 7:**
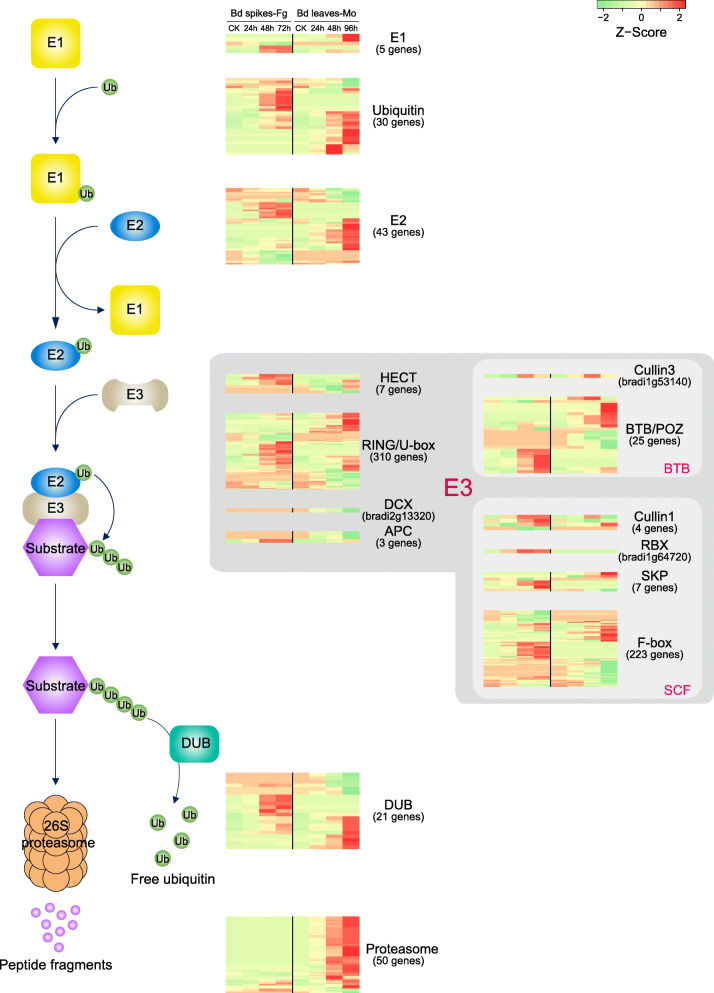
The expression of genes involved the UPS degradation pathway in *Brachypodium* during infection. The expression dynamics of *E1*, *E2*, *E3*, *DEB*, and proteasome genes in the UPS degradation pathway are illustrated with a heatmap. A row in the heatmap indicates a gene, and a column represents a time point of *Brachypodium* infection samples. The gene expression is mapped to color from green to red, representing a lower to a higher abundance of the transcripts

The ubiquitin-proteasome system (UPS) selectively degrades functional proteins carrying the specific ubiquitination signal, thus providing an efficient and rapid strategy to control many different cellular processes, including responses to plant-pathogen interactions [78–81]. The specific ubiquitination signals of the substrate proteins are attached by E1, E2, and E3 conjugation cascades, and the modified substrates are finally degraded by the 26S proteasome [82]. Consistent with GO and MapMan enrichment analyses, a close examination revealed that many genes involved in the UPS pathway were affected (Fig. 7, Table S3). Interestingly, we found that nearly all the proteasome genes were up-regulated in the *M. oryzae* infection, while they were essentially unchanged in the *F. graminearum* infection (Fig. 7). Almost every step of the host defense mechanism is regulated by UPS, such as plant hormone signaling and programmed cell death [78, 79, 83]. In turn, this pathway is frequently targeted by different viral, bacterial, fungal, and oomycete pathogens [83–85]. Therefore, UPS is a central hub as well as a battleground for plant-pathogen interaction. Our findings indicated that gene expression in the UPS is likely manipulated during infection and the up-regulation of proteasome genes is likely switched-off by *F. graminearum*.

## Conclusions

*F. graminearum* and *M. oryzae* are two of the most devastating plant pathogenic fungi. In this study, we studied the transcriptome dynamics of *B. distachyon* infected by these two fungal pathogens. We found that numerous defense-related genes were induced and showed distinct temporal dynamics of expression across the infection time course, including genes encoding RLK, MAPK cascade, phytohormone signaling pathways, and so on. Although the defense systems against the two pathogens were conserved, distinct expression programs and specific defense responses were frequently observed during the infections, suggesting their distinct interaction patterns. In particular, some genes related to JA signaling and the 26S proteasome pathway are likely specifically inhibited or manipulated upon infection by *F. graminearum*. Our study provides valuable insight into the interactions between *B. distachyon* and two important cereal pathogens.

## Methods

### Plant growth

The seeds of *B. distachyon* were sourced from our laboratory (NWAFU-Purdue Joint Research Center, Northwest A&F University). *B. distachyon* ecotype Bd21 was used for *F. graminearum* and *M. oryzae* infection. Bd21 seeds were germinated on damp filter paper at 4 °C in the dark. After one week, they were transferred into pots in a growth chamber at 23 °C ± 2 °C with a 16-h light/8-h darkness photoperiod.

### *F. graminearum* inoculation

The wild-type *F. graminearum* strain PH-1 was routinely cultured on potato dextrose agar (PDA) plates at 25 °C. To prepare the conidial suspensions, cultures were grown for 3–7 days in carboxymethyl cellulose (CMC) medium with shaking at 175 rpm. The resulting conidia were suspended in sterile distilled water at a final concentration of 10^5^ conidia/mL. Flowering heads of Bd21 plants were inoculated with conidial suspensions at the second spikelet from the base. Inoculated spikes were capped with a plastic bag for 24 h to maintain moisture. The samples were collected at 0, 24, 48, and 72 hpi and stored at − 80 °C.

### *M. oryzae* inoculation

The wild-type *M. oryzae* strain Guy11 was cultured on oat meal agar at 22 °C for 7 days. Conidia were harvested with 0.25% (w/v) gelatin and suspended at a final concentration of 10^4^ conidia/mL. The resulting conidial suspension was sprayed evenly on the leaf surface of five-leaf-stage seedlings. Inoculated plants were kept in a moisturizing chamber to maintain 100% air humidity for 24 h and then transferred to a growth chamber at 23 °C ± 2 °C with a 16-h light/8-h darkness photoperiod. The leaf samples were collected at 0, 24, 48, and 96 hpi and stored at − 80 °C.

### RNA-sequencing

The spikelet and leaves inoculated with *F. graminearum* and *M. oryzae*, respectively, were used for RNA-seq analysis. TruSeq Stranded mRNA Sample Prep Kit was used for RNA extraction and library construction. High-throughput sequencing was performed on an Illumina Hiseq 2000 machine at Novogene (Beijing, China).

### Gene expression analysis

The genome of *B. distachyon* [86] was obtained from the MIPS genome database. *F. graminearum* and *M. oryzae* genomes [87, 88] were downloaded from the Fungal Genome Initiative (FGI) site of Broad Institute. After removing low-quality sequences, the clean RNA-seq reads were aligned to genomes with Tophat2 (v2.0.9, “--mate-inner-dist 80 --mate-std-dev 70 --min-intron-length 20 -I 3000 --microexon-search --min-segment-intron 20 --max-segment-intron 3000 --b2-sensitive”) [89]. The program featureCounts (v.1.4.4, “-p -t exon -g gene_id”) [90] was subsequently used to summarize the counts of mapped reads for genomic features. To filter out weakly expressed genes, only those genes with a minimum expression level of 1 count per million in at least 3 of the 24 libraries were included in the analysis.

To assess the biological variability between samples, hierarchical clustering (with the top 1000 genes) was conducted with Pearson correlation distance measure and the pairwise average-linkage method. Multi-dimensional scaling plots were generated by using R package limma [91] with default setting. Differential gene expression analyses were performed for *B. distachyon* with the default setting in R package edgeR [92]. Benjamini-Hochberg method was used to correct for multiple comparisons. Genes were considered differentially expressed between conditions with a false discovery rate (FDR) of below 0.05.

Genes with similar dynamic expression patterns were profiled using the STEM software [43] with a significance of 0.05. The FPKM values of genes were calculated with Cuffnorm (v2.2.1, with default setting). Heatmaps were carried out with heatmap.2 function in the gplots package and hierarchical clustering was conducted with the same distance measure mentioned above.

### Functional enrichment analysis and protein interaction network analysis

Gene Ontology enrichment analysis was performed using the BINGO (v2.44, with default setting) [93] plugin for Cytoscape (v3.2.0, with default setting) [94] and the Ontologizer software (v2.1) [95]. The term-for-term approach was used combined with Benjamini-Hochberg correction for multiple comparisons with a threshold of 0.05. The annotation of *B. distachyon* was obtained from the PLAZA website (http://bioinformatics.psb.ugent.be/plaza). Protein associated networks of *B. distachyon* were constructed in the STRING (v10, with default setting) database.

### Pathway analyses

Pathway analyses were performed with MapMan (v3.5.1) [47]. To identify those BINs significantly affected by the infection, we calculated the induction factor of all genes in a BIN and compared the average induction factor of a BIN to that of all other BINs by the Wilcoxon rank-sum test with FDR < 0.05.

## Supplementary Information

**Additional file 1: Fig. S1.** Global evaluation of the host transcriptomes. (a-b) Multi-dimensional scaling (MDS) plot of the gene expression in *Brachypodium* infected with *F. graminearum* (a) or *M. oryzae* (b), respectively. (c-d) Hierarchical clustering plot of the gene expression in *Brachypodium* infected with *F. graminearum* (c) or *M. oryzae* (d), respectively. Samples were clustered using the Pearson correlation distance measure. **Fig. S2.** Profiles of the differentially expressed genes in *Brachypodium* infected with *F. graminearum* (a) or *M. oryzae* (b), respectively. The horizontal axis indicates different time points. The vertical axis shows the log2 fold change. A gray line represents the expression pattern of a gene, and the bold red/blue line illustrates the average expression pattern of all genes in each cluster. **Fig. S3.** Protein-protein interaction networks of the up-regulated genes in *B. distachyon* infected by *F. graminearum*. The up-regulated genes in each profile were subjected to protein-protein interaction networks analysis. Gene Ontology annotation was used to reveal the function of each submodule of the networks (blue oval). Nodes and edges represent proteins and functional links, respectively. **Fig. S4.** Protein-protein interaction networks of the down-regulated genes in *B. distachyon* infected by *F. graminearum*. The down-regulated genes in each profile were subjected to protein-protein interaction networks analysis. Gene Ontology annotation was used to reveal the function of each submodule of the networks (blue oval). Nodes and edges represent proteins and functional links, respectively. **Fig. S5.** Protein-protein interaction networks of the down-regulated genes in *B. distachyon* infected by *M. oryzae*. The down-regulated genes in each profile were subjected to protein-protein interaction networks analysis. Gene Ontology annotation was used to reveal the function of each submodule of the networks (blue oval). Nodes and edges represent proteins and functional links, respectively

**Additional file 2: Table S1.** Sequencing metrics of the RNA-seq libraries. **Table S2.** Differentially expressed genes of *Brachypodium* highly specific to *F. graminearum* or *M. oryzae* infection. **Table S3.** Metabolic pathway enrichment analysis on *Brachypodium* infected by *F. graminearum* or *M. oryzae*. **Table S4.** Differentially expressed RLK genes of *Brachypodium* during the infection by *F. graminearum* or *M. oryzae*. **Table S5.** Differentially expressed secondary metabolism genes of *Brachypodium* during the infection by *F. graminearum* or *M. oryzae*

## Data Availability

Raw data was deposited in NCBI database under SRA accession number SRR13662575-SRR13662598.

## Abbreviations

FHB: Fusarium head blight
DEGs: differentially expressed genes
hpi: hours post-inoculation
GO: gene ontology
PAMPs: pathogen-associated molecular patterns
RLK: receptor-like kinase
MAPK: Mitogen-activated protein kinase
SA: salicylic acid
JA: jasmonate
ET: ethylene
ABA: abscisic acid
AUX: Auxin
BR: brassinosteroid
PAL: phenylalanine ammonia lyase
UPS: ubiquitin-proteasome system
