# Supplementary material for: Comparative transcriptome analysis reveals distinct gene expression profiles in Brachypodium distachyon infected by two fungal pathogens
Source: BMC Plant Biol. 2021 Jun 30;21:304. doi: 10.1186/s12870-021-03019-0 (PMC8243454; doi:10.1186/s12870-021-03019-0)
Supplement: Supplementary file 1 — Additional file 1: Fig. S1. Global evaluation of the host transcriptomes. (a-b) Multi-dimensional scaling (MDS) plot of the gene expression in Brachypodium infected with F. graminearum (a) or M. oryzae (b), respectively. (c-d) Hierarchical clustering plot of the gene expression in Brachypodium infected with F. graminearum (c) or M. oryzae (d), respectively. Samples were clustered using the Pearson correlation distance measure. Fig. S2. Profiles of the differentially expressed genes in Brachypodium infected with F. graminearum (a) or M. oryzae (b), respectively. The horizontal axis indicates different time points. The vertical axis shows the log2 fold change. A gray line represents the expression pattern of a gene, and the bold red/blue line illustrates the average expression pattern of all genes in each cluster. Fig. S3. Protein-protein interaction networks of the up-regulated genes in B. distachyon infected by F. graminearum. The up-regulated genes in each profile were subjected to protein-protein interaction networks analysis. Gene Ontology annotation was used to reveal the function of each submodule of the networks (blue oval). Nodes and edges represent proteins and functional links, respectively. Fig. S4. Protein-protein interaction networks of the down-regulated genes in B. distachyon infected by F. graminearum. The down-regulated genes in each profile were subjected to protein-protein interaction networks analysis. Gene Ontology annotation was used to reveal the function of each submodule of the networks (blue oval). Nodes and edges represent proteins and functional links, respectively. Fig. S5. Protein-protein interaction networks of the down-regulated genes in B. distachyon infected by M. oryzae. The down-regulated genes in each profile were subjected to protein-protein interaction networks analysis. Gene Ontology annotation was used to reveal the function of each submodule of the networks (blue oval). Nodes and edges represent proteins and functional links, [file 12870_2021_3019_MOESM1_ESM.pdf]

Comparative transcriptome analysis reveals distinct gene expression profiles in *Brachypodium distachyon* infected by two fungal pathogens

Gengrui Zhu[1], Chengyu Gao[1], Chenyu Wu[1], Mu Li[1], Jin-Rong Xu[2], Huiquan Liu[1], Qinhu Wang[1, *]

[1] State Key Laboratory of Crop Stress Biology for Arid Areas and College of Plant Protection, Northwest A&F University, Yangling, Shaanxi 712100, China.

[2] Department of Botany and Plant Pathology, Purdue University, West Lafayette, IN 47907, USA.

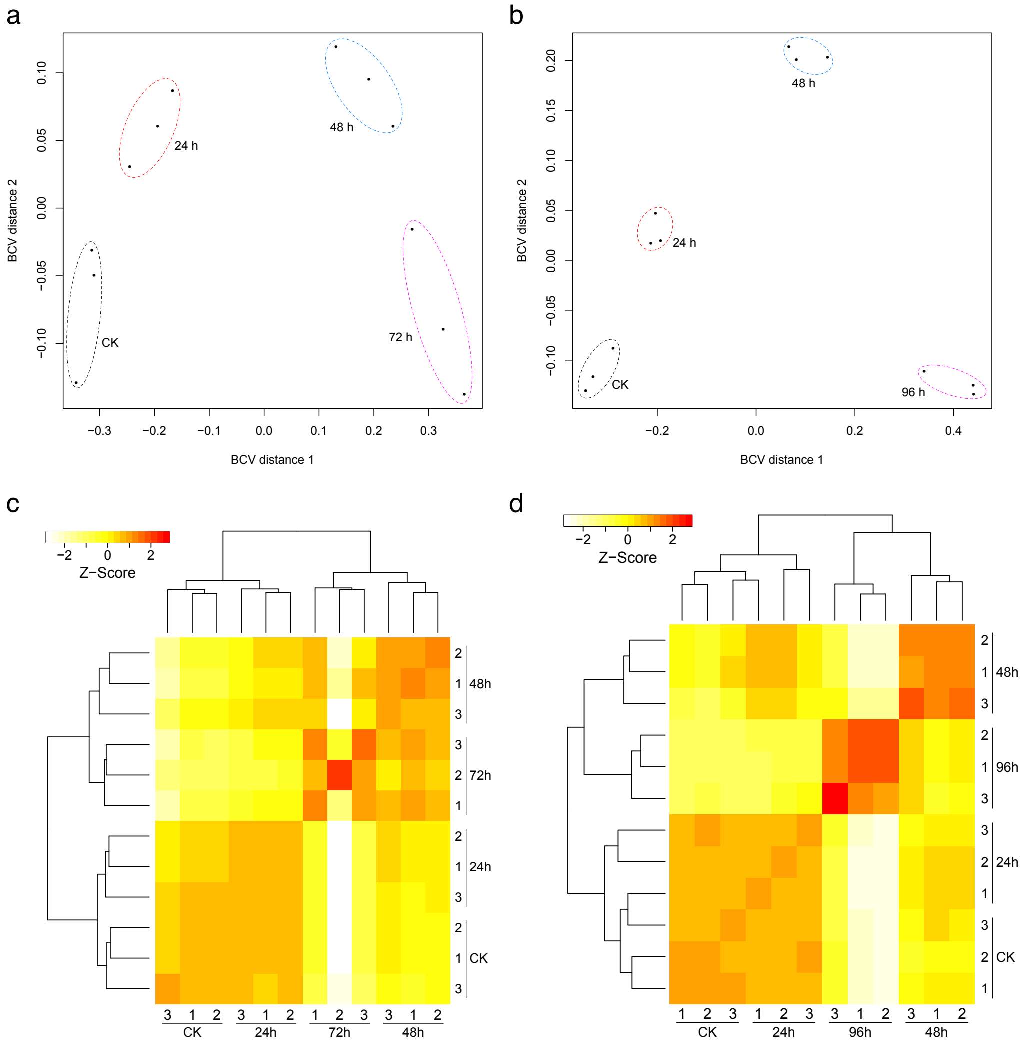

Figure S1. Global evaluation of the host transcriptomes. (a-b) Multi-dimensional scaling (MDS) plot of the gene expression in *Brachypodium* infected with *F. graminearum* (a) or *M. oryzae* (b), respectively. (c-d) Hierarchical clustering plot of the gene expression in *Brachypodium* infected with *F. graminearum* (c) or *M. oryzae* (d), respectively. Samples were clustered using the Pearson correlation distance measure.

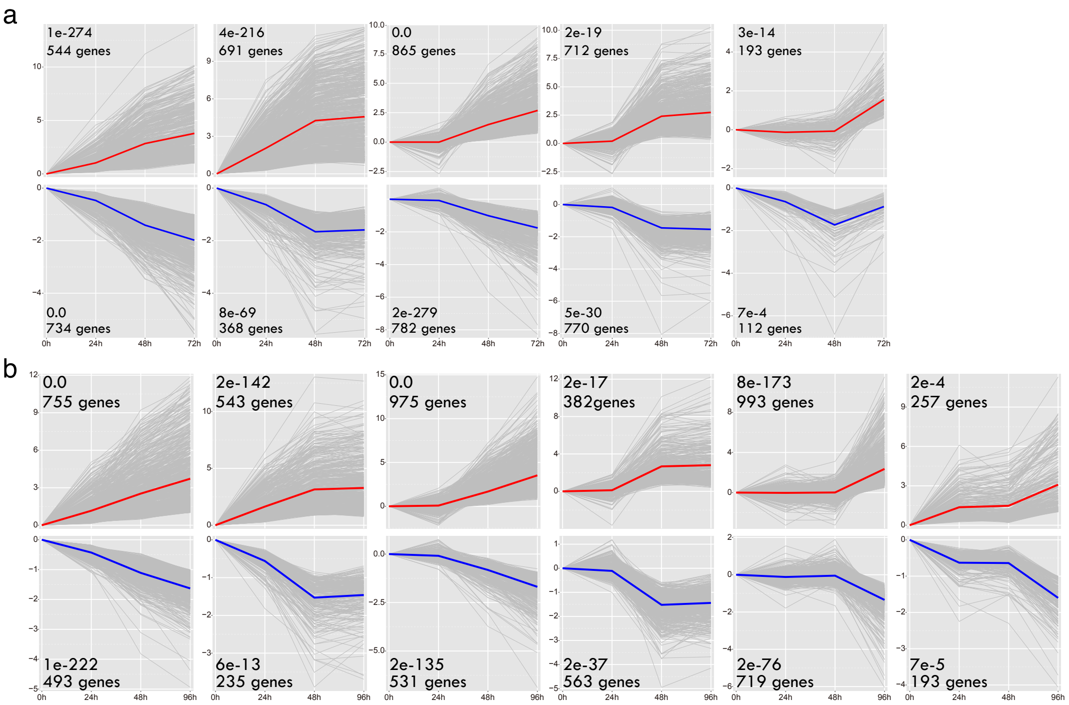

Figure S2. Profiles of the differentially expressed genes in *Brachypodium* infected with *F. graminearum* (a) or *M. oryzae* (b), respectively. The horizontal axis indicates different time points. The vertical axis shows the log2 fold change. A gray line represents the expression pattern of a gene, and the bold red/blue line illustrates the average expression pattern of all genes in each cluster.

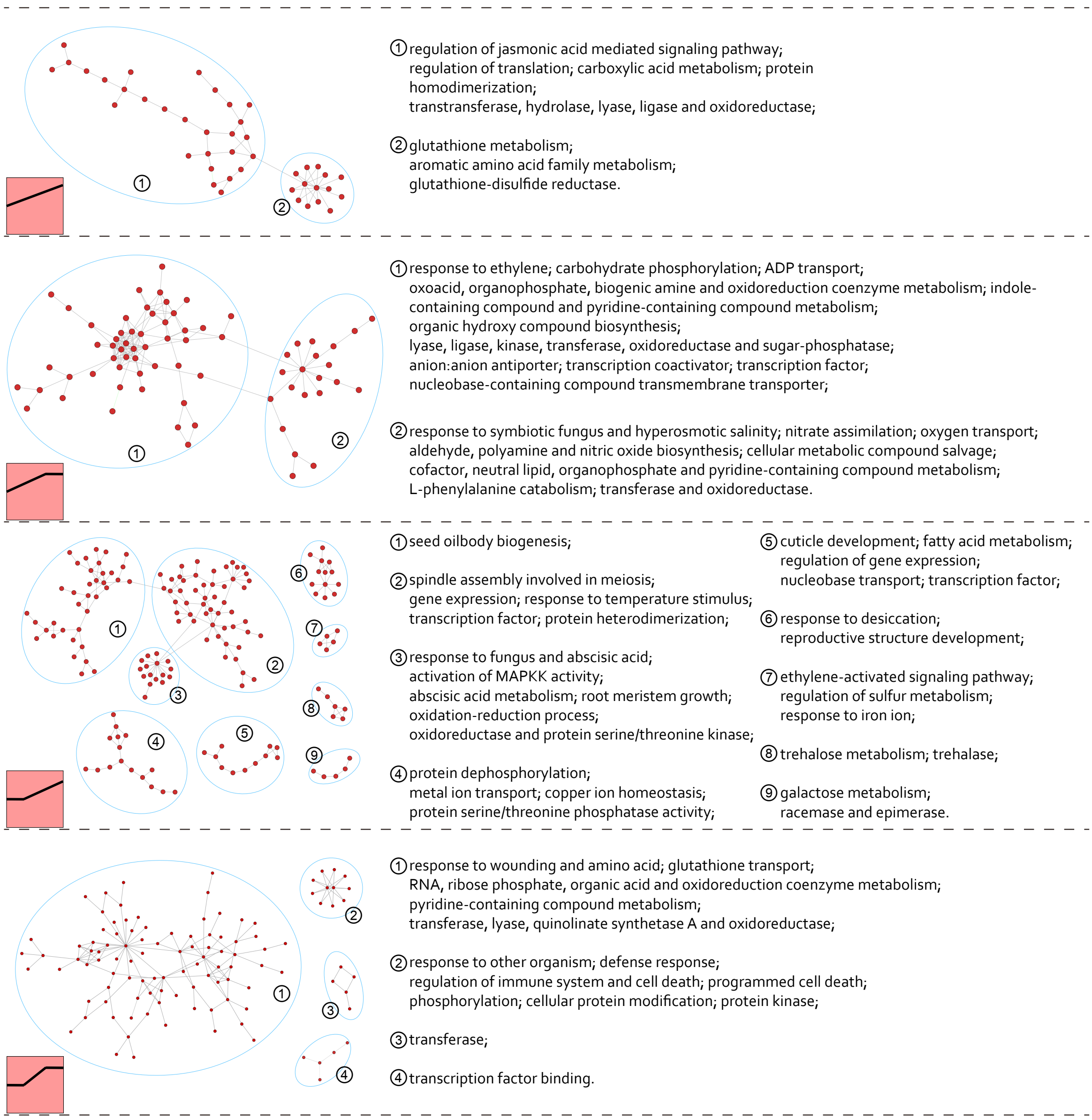

Figure S3. Protein-protein interaction networks of the up-regulated genes in *B. distachyon* infected by *F. graminearum*. The up-regulated genes in each profile were subjected to protein-protein interaction networks analysis. Gene Ontology annotation was used to reveal the function of each submodule of the networks (blue oval). Nodes and edges represent proteins and functional links, respectively.

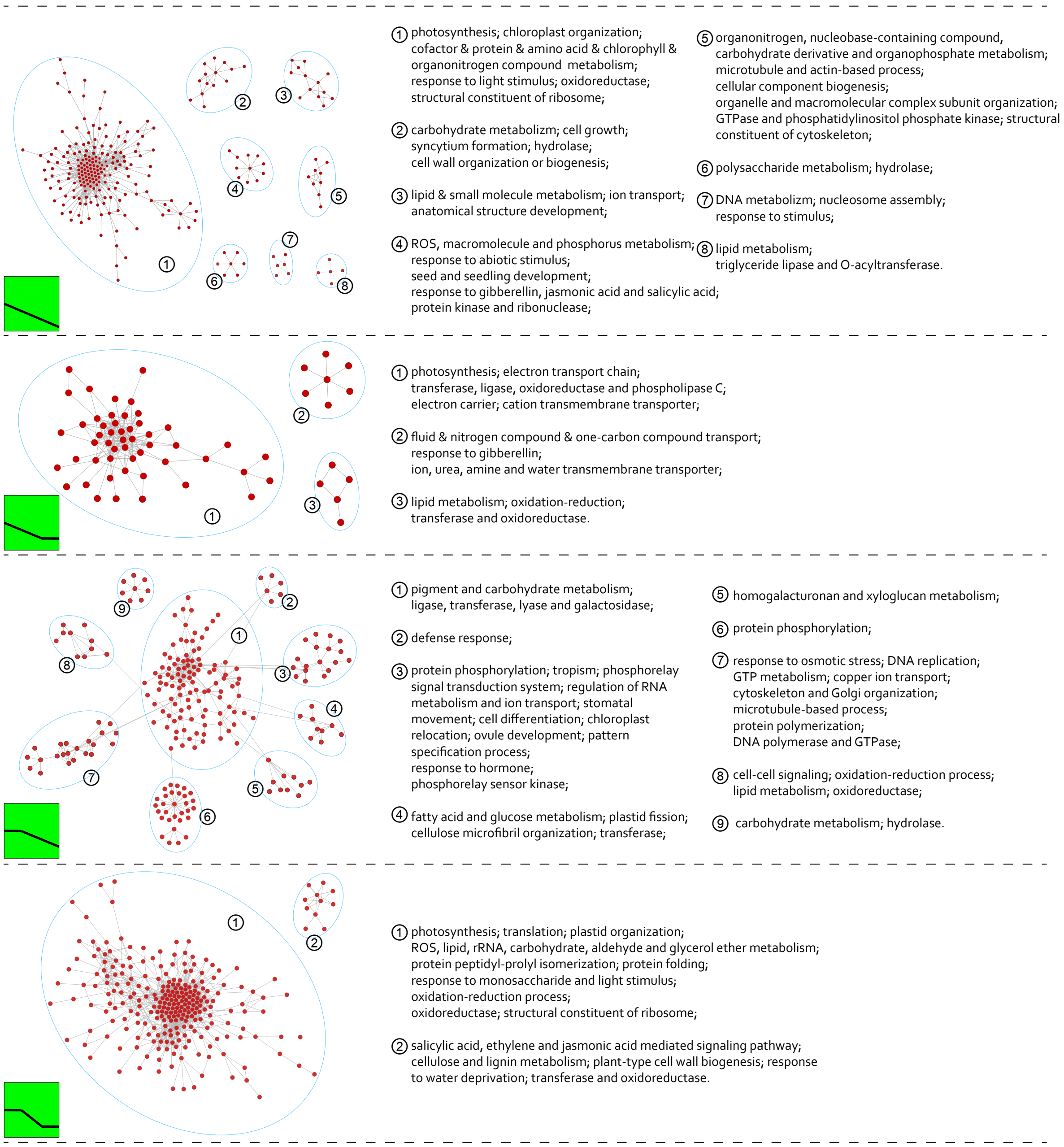

Figure S4. Protein-protein interaction networks of the down-regulated genes in *B. distachyon* infected by *F. graminearum*. The down-regulated genes in each profile were subjected to protein-protein interaction networks analysis. Gene Ontology annotation was used to reveal the function of each submodule of the networks (blue oval). Nodes and edges represent proteins and functional links, respectively.

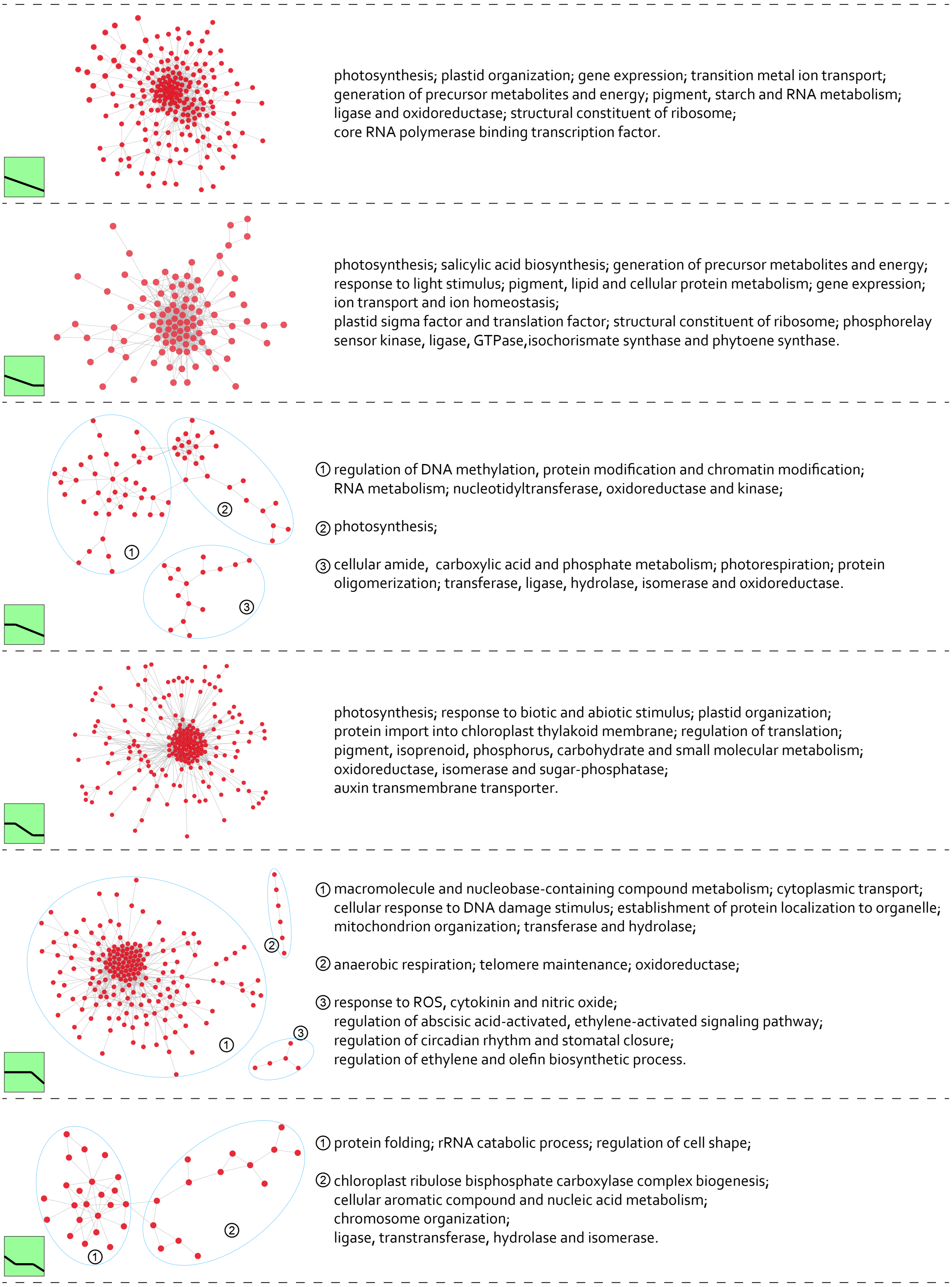

Figure S5. Protein-protein interaction networks of the down-regulated genes in *B. distachyon* infected by *M. oryzae*. The down-regulated genes in each profile were subjected to protein-protein interaction networks analysis. Gene Ontology annotation was used to

reveal the function of each submodule of the networks (blue oval). Nodes and edges represent proteins and functional links, respectively.
